# Does Adhesive Capsulitis of the Shoulder Increase the Risk of Stroke? A Population-Based Propensity Score-Matched Follow-Up Study

**DOI:** 10.1371/journal.pone.0049343

**Published:** 2012-11-19

**Authors:** Chueh-Hung Wu, Yen-Ho Wang, Ya-Ping Huang, Shin-Liang Pan

**Affiliations:** 1 Department of Physical Medicine and Rehabilitation, National Taiwan University Hospital Yun-Lin Branch, Yunlin, Taiwan; 2 Department of Physical Medicine and Rehabilitation, National Taiwan University Hospital and National Taiwan University College of Medicine, Taipei, Taiwan; Universidad Peruana Cayetano Heredia, Peru

## Abstract

**Objectives:**

A previous population-based study reported an increased risk of stroke after the occurrence of adhesive capsulitis of the shoulder (ACS), but there were substantial imbalances in the distribution of age and pre-existing vascular risk factors between subjects with ACS and without ACS, which might lead to a confounded association between ACS and stroke. The purpose of the present large-scale propensity score-matched population-based follow-up study was to clarify whether there is an increased stroke risk after ACS.

**Methods:**

We used a logistic regression model that includes age, sex, pre-existing comorbidities and socioeconomic status as covariates to compute the propensity score. A total of 22025 subjects with at least two ambulatory visits with the principal diagnosis of ACS in 2001 was enrolled in the ACS group. The non-ACS group consisted of 22025, propensity score-matched subjects without ACS. The stroke-free survival curves for these 2 groups were compared using the Kaplan-Meier method. Stratified Cox proportional hazard regression with patients matched on propensity score was used to estimate the effect of ACS on the occurrence of stroke.

**Results:**

During the two-year follow-up period, 657 subjects in the ACS group (2.98%) and 687 in the non-ACS group (3.12%) developed stroke. The hazard ratio (HR) of stroke for the ACS group was 0.93 compared to the non-ACS group (95% confidence interval [CI], 0.83–1.04, P = 0.1778). There was no statistically significant difference in stroke subtype distribution between the two groups (P = 0.2114).

**Conclusions:**

These findings indicate that ACS itself is not associated with an increased risk of subsequent stroke.

## Introduction

Adhesive capsulitis of the shoulder (ACS) is characterized by intense shoulder pain with progressive limitation of shoulder mobility in all planes [1]. Although a minority of cases of ACS have been etiologically linked to previous trauma or surgery of the shoulder, most cases are considered to be idiopathic without a preceding trauma [2]. The etiology and pathogenesis of ACS remains a mystery. Several systemic comorbid diseases, such as diabetes [3], thyroid diseases [4], and hyperlipidemia [5], [6], have been associated with ACS.

Kang et al. [7] performed a population-based study on 10935 subjects with ACS and 32805 subjects without ACS using an insurance database and demonstrated an increased risk of developing stroke after the occurrence of ACS. Compared to the non-ACS subjects, the estimated hazard ratio of stroke for the ACS patients was 1.22 after adjusting for demographic characteristics, diabetes, hypertension, and heart diseases [7]. On the basis of these findings, clinicians should be alert to the possibility of cerebrovascular accident when treating patients with ACS since stroke is a highly disabling or even fatal disease resulting in enormous social burden. However, in their study, there was a substantial imbalance in the distribution of demographic characteristics and vascular risk factors between the ACS and non-ACS groups. The ACS group was significantly older and had a 1.5-fold higher prevalence of diabetes and hyperlipidemia than the non-ACS group. Such a significant imbalance may not be effectively overcome by adjustment for these potential confounding factors in multiple regression analysis, especially when the estimated increase in stroke risk associated with ACS is only modest. Since age, diabetes, and hyperlipidemia are well-known risk factors for stroke [8]–[11], this raises the possibility that the modest association between ACS and stroke may be attributed to the imbalance in these risk factors, rather than ACS itself. One way of answering this question is to match age, sex, and various clinical characteristics between subjects with and without ACS [12], [13]. Propensity score matching methods are increasingly being used in observational studies to reduce bias [14], [15]. We therefore performed a large-scale propensity score-matched follow-up study to investigate whether there is an increased risk of stroke after the occurrence of ACS.

## Methods

### Data Source

The data used in this study were obtained from the complete National Health Insurance (NHI) claim database in Taiwan for the period 2000 to 2003. The NHI program has been implemented in Taiwan since 1995, and the coverage rate was 96% of the whole population in 2000 and 97% at the end of 2003, at which time more than 21.9 million inhabitants were enrolled. It should be noted that the rationale for using the NHI database after 2000 is that, from Jan 1, 2000, according to the rules of the Bureau of NHI, the NHI claim data were all encoded using the standardized International Classification of Disease, 9^th^ Revision, Clinical Modification (ICD-9-CM). To keep individual information confidential in order to satisfy regulations on personal privacy in Taiwan, all personal identification numbers in the data were encrypted by converting the personal identification numbers into scrambled numbers before data processing. Because the database used consists of de-identified secondary data released for research purposes, this principle complies with the Personal Information Protection Act in Taiwan, and this study was exempt from full review by the Institutional Review Board.

### Study Subjects and Design

We used a cohort study design to investigate the effect of ACS on the risk for developing subsequent stoke. To control for potential confounding from imbalance in clinical characteristics, we used propensity score matching to create comparable cohorts between patients with and without ACS [16], [17]. The study population consisted of an ACS group and a non-ACS group, both selected from Taiwanese residents in the complete NHI claim database for 2001, in which more than 21.6 million persons were registered. The ACS group consisted of subjects aged 40 years or older who had received a principal diagnosis of ACS (ICD-9-CM code 726.0) during ambulatory medical care visits between January 1, 2001 and December 31, 2001. The index visit was defined as the first ambulatory visit during which a principal diagnosis of ACS was made. To maximize case ascertainment, only patients who had at least 2 ambulatory visits (including the index visit) with the principal diagnosis of ACS in this period were considered for inclusion in the ACS group (n = 36790). The exclusion criteria for the recruitment of subjects into the ACS group were: (1) a previous diagnosis of ACS (ICD-9-CM code 726.0) during 2000 (n = 9616) so as to increase the likelihood of identifying only new incident ACS cases in 2001; (2) a previous diagnosis of any type of stroke (ICD-9-CM codes 430–438) before the index ambulatory care visit (n = 3986); (3) a previous diagnosis of rheumatic disorders (ICD-9-CM codes 710, 714, and 720) before the index visit (n = 2390); and (4) a previous diagnosis of fracture or dislocation of the shoulder (ICD-9-CM codes 810, 811, 812, and 831) before the index visit (n = 1612). A total of 22235 subjects was identified in the ACS group.

### Covariates and Propensity Score Matching

The information of pre-existing comorbidities, including diabetes (ICD-9-CM code 250), hypertension (ICD-9-CM codes 401–405), hyperlipidemia (ICD-9-CM code 272), coronary heart disease (ICD-9-CM codes 410–414 and 429.2), chronic rheumatic heart disease (ICD-9-CM codes 393–398), and other types of heart disease (ICD-9-CM codes 420–429), were acquired by tracking all the ambulatory medical care and inpatient records in the NHI database in the year before the index visit. The case ascertainment for these medical comorbidities was defined from ≧1 hospital discharge or ≧2 ambulatory visits with a relevant principal or secondary diagnosis code. Previous studies have suggested that the risk of stroke may be affected by socioeconomic status such as geographical regions, levels of urbanization, and income levels [18], [19]. Therefore, these factors are also taken into account as variables in assessing the risk of stroke. The information of the geographical location of residency of each subject was obtained from the population household registry. The geographical location of residency was classified into Northern, Central, Eastern, and Southern Taiwan. In accordance with Taiwan National Health Research Institute publications [20], urbanization levels in Taiwan are classified into 7 strata, with level 1 referring to the “most urbanized” and level 7 referring to the “least urbanized” communities. However, since there were relatively small number of subjects in levels 5, 6, and 7, these 3 levels were merged into a single group and labeled as level 5. For the income-level, we used the insured payroll-related amount as a proxy for income (0, NT$1 to NT$15840, NT$15841 to NT$25000, NT$25001; NT$ indicates new Taiwan dollar). Note that we selected NT$15840 as the first cutoff point of income level because this is the government-stipulated minimum wage for full-time employees in Taiwan. Since the household registry information is not available in 210 subjects out of the 22235 subjects in the ACS group, these 210 subjects were excluded from the analysis. The final ACS group consisted of 22025 subjects.

The non-ACS group was taken from the remaining subjects in the same 2001 NHI claim database without a diagnosis of ACS. We assigned the first ambulatory medical care visit during 2001 as the index ambulatory visit. The exclusion criteria for recruiting subjects into the non-ACS group were: (1) a previous diagnosis of ACS before the index visit; (2) a previous diagnosis of any type of stroke before the index visit; (3) a previous diagnosis of rheumatic disorders before the index visit; and (4) a previous diagnosis of fracture or dislocation of the shoulder before the index visit. The information of pre-existing co-morbidities and socioeconomic status were acquired using the same methods described above. Because the number of subjects in the NHI database is very large, we used a two-stage method to select the propensity score-matched non-ACS group. For each subject in the ACS group, we first randomly sampled 20 age and sex-matched non-ACS subjects who met the abovementioned criteria. A total of 440500 non-ACS subjects was initially sampled. In the second stage, a logistic regression model including age, sex, pre-existing co-morbidities and socioeconomic status as covariates was used to predict the probability (i.e. propensity score) of ACS. An 8-to-1 greedy matching algorithm [16] was then used to identify a unique matched control from the 440500 non-ACS subjects for each ACS patient according to the propensity score. A total of 22025 subjects was selected in the propensity score-matched non-ACS group.

**Table 1:** Demographic characteristics and comorbid medical disorders for the adhesive capsulitis (ACS) and Non-ACS groups before propensity score matching.

Variables	ACS group (N = 22025)	Non-ACS group (N = 440500)	
	N(%)	N(%)	*p* value
Sex(women)	13158 (59.7)	263160 (59.7)	1.0000
Age(years)			1.0000
40–49	6057 (27.5)	121140 (27.5)	
50–59	6880 (31.2)	137600 (31.2)	
60–69	5241 (23.8)	104820 (23.8)	
70–79	3267 (14.8)	65340 (14.8)	
80+	580 (2.6)	11600 (2.6)	
Diabetes(yes)	3082 (14.0)	44677 (10.1)	<.0001
Hypertension(yes)	5577 (25.3)	102144 (23.2)	<.0001
Hyperlipidemia(yes)	2342 (10.6)	31549 (7.2)	<.0001
Coronary heart disease(yes)	2207 (10.0)	33743 (7.7)	<.0001
Chronic rheumatic heart disease(yes)	130 (0.6)	2128 (0.5)	0.0260
Other heart disease(yes)	1707 (7.7)	26223 (6.0)	<.0001
Monthly income			<.0001
NT$0	6153 (27.9)	122030 (27.7)	
NT$1– NT$15840	3293 (15.0)	54361 (12.3)	
NT$15840–NT$25000	8373 (38.0)	190093 (43.2)	
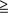 NT$25000	4206 (19.1)	74016 (16.8)	
Urbanization level			<.0001
1	4597 (20.9)	85130 (19.3)	
2	2747 (12.5)	48930 (11.1)	
3	6587 (29.9)	113948 (25.9)	
4	3122 (14.2)	67855 (15.4)	
5	4972 (22.6)	124637 (28.3)	
Geographic region			<.0001
Northern	11645 (52.9)	194583 (44.2)	
Central	3517 (16.0)	81070 (18.4)	
Southern	6313 (28.7)	151878 (34.5)	
Eastern	550 (2.5)	12969 (2.9)	
Propensity score	0.051±0.014	0.047±0.013	<.0001

Data are expressed as N (%) or mean±SD.

US $1 = NT $34 in 2001.

**Table 2:** Demographic characteristics and comorbid medical disorders for the adhesive capsulitis (ACS) and Non-ACS groups after propensity score matching.

Variables	ACS group (N = 22025)	Non-ACS group (N = 22025)	
	N(%)	N(%)	*p* value
Sex(women)	13158 (59.7)	13160 (59.7)	0.9845
Age (years)			0.3039
40–49	6057 (27.5)	6117 (27.8)	
50–59	6880 (31.2)	6910 (31.4)	
60–69	5241 (23.8)	5077 (23.1)	
70–79	3267 (14.8)	3295 (15.0)	
80+	580 (2.6)	626 (2.8)	
Diabetes(yes)	3082 (14.0)	3003 (13.6)	0.2753
Hypertension(yes)	5577 (25.3)	5525 (25.1)	0.5682
Hyperlipidemia(yes)	2342 (10.6)	2285 (10.4)	0.3757
Coronary heart disease(yes)	2207 (10.0)	2229 (10.1)	0.7276
Chronic rheumatic heart disease(yes)	130 (0.6)	134 (0.6)	0.8050
Other heart disease(yes)	1707 (7.7)	1712 (7.8)	0.9291
Monthly income			0.7971
NT$0	6153 (27.9)	6204 (28.2)	
NT$1–NT$15840	3293 (15.0)	3227 (14.7)	
NT$15840–NT$25000	8373 (38.0)	8356 (37.9)	
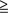 NT$25000	4206 (19.1)	4238 (19.2)	
Urbanization level			0.9117
1	4597 (20.9)	4560 (20.7)	
2	2747 (12.5)	2799 (12.7)	
3	6587 (29.9)	6630 (30.1)	
4	3122 (14.2)	3097 (14.1)	
5	4972 (22.6)	4939 (22.4)	
Geographic region			0.7142
Northern	11645 (52.9)	11721 (53.22)	
Central	3517 (16.0)	3494 (15.9)	
Southern	6313 (28.7)	6235 (28.3)	
Eastern	550 (2.5)	575 (2.6)	
propensity score	0.051±0.014	0.051±0.014	0.9924

Data are expressed as N (%) or mean±SD.

US $1 = NT $34 in 2001.

### Outcome

All ambulatory medical care records and inpatients records for each subject in the propensity score-matched ACS and non-ACS groups were tracked from their index visit for 2 years and mortality data for the subjects who died during the follow-up were obtained from the national mortality registry. The date of the first occurrence of a principal diagnosis of stroke (ICD-9-CM codes 430–437) within the follow-up period was defined as the primary endpoint. The case ascertainment for stroke required ≧1 hospital discharge or ≧2 ambulatory medical care visits with the principal diagnosis of stroke. All subjects were followed from the index visit to the first occurrence of stroke, death, or end of follow-up.

### Statistical Analysis

The Chi-squared test and student’s t test were used to examine differences in demographic variables, comorbid medical disorders and propensity scores between the ACS and non-ACS groups. The stroke-free survival curves of the propensity-score matched ACS and non-ACS groups were generated using the Kaplan-Meier method and the difference in survival between these two groups was assessed using the log-rank test. Stratified Cox proportional hazard regression with patients matched on propensity score was used to estimate the effect of ACS on the occurrence of stroke. An alpha level of 0.05 was considered statistically significant for all analyses. The analyses were performed using SAS 9.2 software (SAS Institute, Cary, NC).

## Results

Table 1 shows the demographic and clinical characteristics of the ACS and non-ACS groups before propensity score matching. The ACS group had a higher prevalence of certain pre-existing medical comorbidities, including hypertension (P<0.0001), hyperlipidemia (P<0.0001), chronic rheumatic heart disease (P = 0.0260), coronary heart disease (P<0.0001), and other heart disease (P<0.0001) than the non-ACS group. There were also significant differences in the distribution of monthly income, urbanization level, and geographic region between the ACS and non-ACS groups. The ACS groups had higher propensity score than the non-ACS group (P<0.0001). After propensity score matching, the matched cohorts were well-balanced in terms of all observed covariates (Table 2). There was no statistically significant difference in all the baseline characteristics between the ACS group and matched non-ACS group (Table 2).

**Figure 1:**
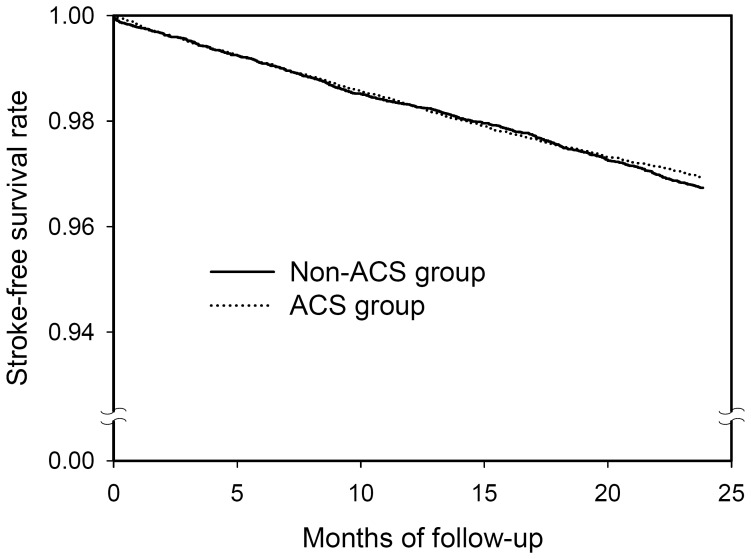
Two-year stroke-free survival rates for the propensity score-matched adhesive capsulitis (ACS) group (dotted line) and the non-ACS group (solid line).

The number of stroke events and the hazard ratios (HR) of stroke during the two-year follow-up period for the two propensity score-matched groups are presented in Table 3. Of the 22025 subjects with ACS, 657 (2.98%) developed stroke compared to 687 (3.12%) of the 22025 subjects in the non-ACS group. The HR of stroke for patients with ACS was 0.93 (95% confidence interval [CI], 0.83–1.04, P = 0.1778). The two-year stroke-free survival rates for the two groups are shown in Figure 1. No significant difference in stroke-free survival rate was seen between the two groups (P = 0.2818).

We also compared the distribution of stroke subtypes between the two matched groups (Table 3). Of the 657 stroke events in the ACS group, 67 (10.2%) were hemorrhagic stroke (ICD-9-CM code 430–432), 344 (52.4%) ischemic stroke (ICD-9-CM code 433–435), and 246 (37.4%) other types of stroke (ICD-9-CM code 436–437), while the corresponding figures for the 687 stroke events in the non-ACS group were 53 (7.7%), 383 (55.8%), and 251 (36.5%). There was no statistically significant difference in stroke subtype distribution between the two groups (P = 0.2114).

**Table 3:** Number of stroke events, the hazard ratio of stroke, and the distribution of stroke subtypes for the matched adhesive capsulitis (ACS) and non-ACS groups.

Variables	ACS groupN = 22025	Non-ACSgroupN = 22025	*p* value
Stroke events, N (%)	657 (2.98)	687 (3.12)	
Hazard Ratio (95% CI)	0.93 (0.83–1.04)	1.00	0.1778
Distribution of subtypes*			0.2114
Hemorrhagic stroke, N (%)	67 (10.2)	53(7.7)	
Ischemic stroke, N (%)	344 (52.4)	383(55.8)	
Other types of stroke, N (%)	246 (37.4)	251(36.5)	

* Hemorrhagic stroke (ICD-9-CM codes 430–432), ischemic stroke (ICD-9-CM codes 433–435), and other types of stroke (ICD-9-CM codes 436–437).

## Discussion

Kang et al. [7] analyzed the NHI research database and reported a modestly increased risk of stroke after occurrence of ACS (adjusted HR = 1.22, 95% CI, 1.06–1.40). In the present large-scale population-based follow-up study, we attempted to replicate the previously reported positive association between ACS and stroke. However, using the complete NHI database, we found that occurrence of ACS was not associated with an increased stroke risk after applying propensity score-matching to balance the baseline characteristics between subjects with and without ACS (HR = 0.93, 95% CI, 0.83–1.04), and there was no significant difference in stroke-free survival rate between the ACS and non-ACS groups. We think this discrepancy is mainly attributed to the substantial imbalance in the distribution of age, diabetes, and hyperlipidemia in the study of Kang et al, which did not match these variables in the ACS and non-ACS subjects. As ACS has been associated with age, diabetes, and hyperlipidemia [3], [21], [22], it might be expected that, without matching, subjects with ACS would be older and have a higher prevalence of diabetes and hyperlipidemia than those without ACS, as seen in the study of Kang et al. Moreover, age, diabetes, and hyperlipidemia are known risk factors for stroke [8]–[11]. Consequently, an imbalance in the baseline demographic and comorbidity variables would lead to a confounded association between ACS and stroke. Although multiple regression analysis has been applied to the adjustment for potential confounders in observational studies, the confounding may not be completely overcome by covariate adjustment in multiple regression analysis due to the following concerns. First, multiple regression analysis commonly assumes linearity of the effects of confounders. However, the true effect may be a non-linear form such as quadratic or exponential [14]. Second, unless the confounders are measured without error, the confounding cannot be completely removed simply by adjusting for covariates in multiple regression [14]. Matching is an alternative method to control for confounding. However, an important drawback is the difficulty in finding close matches as the number of variables for matching increases. Propensity scoring summarizes all measured potential confounders into a single composite score [15]. Matching on propensity score will be similar to matching on all the included covariates used for computing the propensity score. As shown in Table 2, there was no significant difference in all demographic and comorbidities variables after matching. By applying propensity score matching, we minimized the potential confounding effects of these variables and found that occurrence of ACS was not related to an increased risk of subsequent stroke.

In our study, there was no significant difference in the distribution of stroke subtypes between the ACS and non-ACS groups (Table 3). This finding is different from that of Kang et al [7], who reported a significantly higher risk of ischemic stroke in subjects with ACS than those without ACS. It has been suggested that persons with advanced age and diabetes are prone to developing ischemic stroke, rather than hemorrhagic stroke [23], [24], which may account for the predisposition of ACS subjects to develop ischemic stroke reported by Kang et al, as the ACS group in their study included more subjects with advanced age and diabetes. By matching demographic and comorbidities variables, we demonstrated that there was no predisposition of ACS patients to develop ischemic stroke. This result is consistent with our above finding that ACS is not associated with an increased risk of stroke.

Before matching on propensity scores (Table 1), the ACS group had higher prevalence of pre-existing major vascular risk factors including diabetes, hypertension, and hyperlipidemia than the non-ACS group. Although diabetes and hyperlipidemia have been associated with ACS [3], [5], [25], the mechanisms underlying such association are not clear. Diabetes is considered to be a chronic inflammatory condition with elevated inflammatory markers [26], [27]. In addition, increased expression of vascular endothelial growth factor in the synovium has been found in patients with diabetes [28]. Therefore, the association between diabetes and ACS may be explained, at least in part, by a diabetes-related chronic inflammatory process with increased growth factor expression, which, in turn, leads to joint synovitis and subsequent capsular fibrosis.

Several strengths of this study should be addressed. First, this study used a longitudinal population-based database, which enabled us to identify all incident strokes and evaluate the temporal relationship between ACS and stroke. Second, we used propensity score matching to minimize the potential confounding effects of all the included covariates. Third, the large study population (22025 ACS subjects and 22025 matched non-ACS subjects) can provide adequate statistical power to minimize the chance of false-negative results on the association between ACS and stroke.

Nevertheless, this study is subject to several possible limitations. First, the diagnosis of diabetes, ACS, stroke, and medical comorbidities was entirely determined using the ICD codes from the NHI claim database and there may be concern about the diagnostic accuracy of the database. However, the Bureau of the NHI has formed different audit committees that randomly sample the claim data from every hospital and review charts on a regular basis to verify the diagnostic validity and quality of care. Accordingly, the NHI claim database is an established research database and has been used in various biomedical research fields [29], [30]. Second, in the ambulatory medical setting, the diagnosis of ACS is usually made on a clinical basis without using gold standard diagnostic tools, such as an arthrogram or arthroscopy. Nevertheless, it has been suggested that most cases of ACS can be effectively diagnosed through adequate history taking and physical examination [2]. Third, due to the inherent limitation of the NHI database, information was lacking regarding lifestyle factors, such as smoking, alcohol consumption, physical inactivity, and obesity, which may affect the interpretation of our findings. However, since lifestyle factors have not been identified as risk factors for ACS and since no significant association between ACS and stroke was found in our study, we believe that lifestyle factors are not likely to confound the relationship between ACS and stroke.

In conclusion, the present large-scale population-based propensity score-matched study showed that occurrence of ACS was not linked to an increased risk of developing stroke. Our findings suggest that the previously reported positive association between ACS and stroke may result from the confounding effects of age and pre-existing comorbidities.
